# Doxycycline-Induced Expression of Transgenic Human Tumor Necrosis Factor α in Adult Mice Results in Psoriasis-like Arthritis

**DOI:** 10.1002/art.38026

**Published:** 2013-08-26

**Authors:** Eugen Retser, Tanja Schied, Boris V Skryabin, Thomas Vogl, Janos M Kanczler, Nina Hamann, Anja Niehoff, Sven Hermann, Michel Eisenblätter, Lydia Wachsmuth, Thomas Pap, Peter L E M van Lent, Karin Loser, Johannes Roth, Frank Zaucke, Stephan Ludwig, Viktor Wixler

**Affiliations:** 1 ZMBE and University Hospital MuensterMuenster, Germany; 2 ZMBE, IZKF, and University Hospital MuensterMuenster, Germany; 3 University Hospital MuensterMuenster, Germany; 4 University of Southampton Medical SchoolSouthampton, UK; 5 German Sport University CologneCologne, Germany; 6 University of MuensterMuenster, Germany; 7 Radboud University Nijmegen Medical CentreNijmegen, The Netherlands; 8 University of CologneCologne, Germany

## Abstract

**Objective:**

To generate doxycycline-inducible human tumor necrosis factor α (TNFα)–transgenic mice to overcome a major disadvantage of existing transgenic mice with constitutive expression of TNFα, which is the limitation in crossing them with various knockout or transgenic mice.

**Methods:**

A transgenic mouse line that expresses the human TNFα cytokine exclusively after doxycycline administration was generated and analyzed for the onset of diseases.

**Results:**

Doxycycline-inducible human TNFα–transgenic mice developed an inflammatory arthritis– and psoriasis-like phenotype, with fore and hind paws being prominently affected. The formation of “sausage digits” with characteristic involvement of the distal interphalangeal joints and nail malformation was observed. Synovial hyperplasia, enthesitis, cartilage and bone alterations, formation of pannus tissue, and inflammation of the skin epidermis and nail matrix appeared as early as 1 week after the treatment of mice with doxycycline and became aggravated over time. The abrogation of human TNFα expression by the removal of doxycycline 6 weeks after beginning stimulation resulted in fast resolution of the most advanced macroscopic and histologic disorders, and 3–6 weeks later, only minimal signs of disease were visible.

**Conclusion:**

Upon doxycycline administration, the doxycycline-inducible human TNFα–transgenic mouse displays the major features of inflammatory arthritis. It represents a unique animal model for studying the molecular mechanisms of arthritis, especially the early phases of disease genesis and tissue remodeling steps upon abrogation of TNFα expression. Furthermore, unlimited crossing of doxycycline-inducible human TNFα–transgenic mice with various knockout or transgenic mice opens new possibilities for unraveling the role of various signaling molecules acting in concert with TNFα.

Tumor necrosis factor α (TNFα) is the prototype of a proinflammatory cytokine that is mainly secreted by activated macrophages, but also by keratinocytes, fibroblasts, and endothelial cells. It plays a key role in activation of immune cells during the acute phase of inflammation, but it also regulates fundamental cell responses such as proliferation, differentiation, and apoptosis (1). When dysregulated, TNFα displays several pathologic activities resulting in the development of acute and chronic pathologies, among which psoriatic arthritis (PsA) and rheumatoid arthritis (RA) are the most prominent (2–4). In addition to attraction and activation of immune cells, TNFα also activates synovial fibroblasts, keratinocytes, and osteoclasts, which in turn, secrete further cytokines, chemokines, and alarmins, thus potentiating a proinflammatory state and tissue damage. Furthermore, the alarmins S100A8 and S100A9 have been shown to act in a concerted manner with TNFα. S100A8 and S100A9 up-regulate TNFα expression and TNFα up-regulates S100A8 and S100A9 expression in a functional feedback loop, and both S100A8 and S100A9 are involved in induction of matrix metalloproteinases (MMPs) (5,6), contributing significantly to inflammation, cartilage damage, and bone resorption (7–9).

Considering the great importance of TNFα for the induction and progression of arthritis, it is not surprising that several mouse models overexpressing the TNFα cytokine have been established. The first was generated by Keffer et al in 1991 (10). This transgenic mouse contains the complete genomic sequence of the human TNFα gene. Only the 3′-noncoding region was replaced by the 3′-untranslated region (3′-UTR) of the human β-globin gene. These mice constitutively express the human TNFα cytokine and, 10 weeks after birth, develop a severe form of RA that affects primarily ankle joints. Other transgenic models soon followed. They constitutively express either a human or a mouse TNFα transgene with a deleted or modified 3′-UTR. These transgenic mice, as well as the tristetraprolin-knockout mouse, all developed arthritic diseases of differing severity and allowed the deciphering of many molecular details underlying their development (4,11). However, TNFα-transgenic mice usually also have impaired fertility (12). This dysfunction presents a serious hindrance in crossing them with other animals to gain more insight into the molecular mechanisms of arthritic diseases or other disorders. To overcome this problem, we generated a doxycycline-inducible human TNFα–transgenic mouse (also called an ihTNFtg mouse) in which the expression of the human TNFα gene is under the control of a tetracycline (Tet)–responsive promoter.

## MATERIALS AND METHODS

### Animal studies

The details of generating the doxycycline-inducible human TNFα–transgenic mouse are described at http://zmbe.uni-muenster.de/vwixler/retser.zip. The animals were maintained under pathogen-free conditions, and all experiments were performed on mice homozygous for both the Tg_rtTA2S transgene and the human TNFα transgene. Genotyping was performed with TaqMan quantitative reverse transcription–polymerase chain reaction (qRT-PCR), using genomic DNA from tail biopsy samples. To induce expression of the human TNFα transgene, 6-week-old mice were kept on drinking water containing doxycycline (Sigma) and 5% sucrose for the times indicated. The water was protected from light and exchanged every 3 days.

Clinical assessments were performed weekly during the doxycycline stimulation of the mice. Paw swelling was graded from 0 to 3, and grip strength was graded from 0 to –3 as previously described (13). The amount of human TNFα cytokine or mouse S100A8 and S100A9 proteins in the serum of the mice was analyzed by enzyme-linked immunosorbent assay (ELISA) as described previously (14).

### In vivo imaging

Magnetic resonance imaging (MRI) was performed with a 9.4T small animal MR scanner with a helium-cooled Cryoprobe (Bio-Spec 94/20; Bruker BioSpin MRI) and using ParaVision 5.1 software (Bruker BioSpin MRI). Anesthetized mice were placed in the animal cradle in a supine position with the fore limbs taped above the nose cone. Isotropic 3-dimensional fast low-angle shoot sequence images (repetition time 30 msec, echo time 4.2 msec, flip angle 15°, spatial resolution 52 μm^3^) were acquired pre- and postbolus intravenous (IV) application of Magnevist (0.5 mmoles/ kg Gd; Bayer Healthcare). Total scan time per animal was ∼1 hour.

Leukocytes as a driving force of inflammatory processes are highly glucose-avid, which allows the visualization of local inflammation by ^18^F-fluorodeoxyglucose (^18^F-FDG). Doxycycline-inducible human TNFα–transgenic mice were injected IV with 10 MBq ^18^F-FDG, and 1 hour later, a positron emission tomography (PET) list-mode scan was performed for 15 minutes using a 32-module quadHIDAC scanner (Oxford Positron Systems) dedicated to small animal imaging. The scanner has an effective resolution of 0.7 mm (full width at half maximum) in the transaxial and axial directions when using an iterative resolution recovery reconstruction algorithm. To noninvasively assess bone turnover, the distribution of ^99m^Tc-labeled methylene diphosphonate (MDP) was measured by single-photon–emission computed tomography (SPECT) (NanoSPECT/CT; Mediso) 1 hour post–IV injection of MDP.

High-resolution micro-CT scanning was performed with a μCT 35 scanner (Scanco Medical). Dissected fore paws were scanned with an isotropic voxel size of 7 μm, 70 kVp tube voltage, 114 mA tube current, 200 msec integration time, and frame averaging of 1. Distal interphalangeal (DIP) joints were scanned with an isotropic voxel size of 3.5 μm, 70 kVp tube voltage, 114 mA tube current, 400 msec integration time, and frame averaging of 2. A constrained Gaussian filter (support = 2, σ = 1.2) was used to remove noise in the original volume data. Bone tissue was segmented using a global thresholding algorithm (paws, 23.0% for 0 weeks doxycycline and 18.0% for 6 weeks doxycycline; DIP joints, 24.0% for 0 weeks doxycycline and 19.0% for 6 weeks doxycycline).

### RNA isolation, complementary DNA (cDNA) synthesis, and TaqMan qRT-PCR

Mouse organs were collected, minced, and immediately transferred into RNAlater solution (Ambion). After overnight incubation at 4°C, they were homogenized, and the RNA was isolated by a Qiagen RNeasy kit. The RNA integrity was controlled by a Bioanalyzer 2100 system (Agilent Technologies) and transcribed into cDNA using a high-capacity cDNA reverse transcription kit from Applied Biosystems. Levels of messenger RNA (mRNA) expression were determined by TaqMan qRT-PCR using a LightCycler 480 II instrument (Roche Diagnostics). Each cDNA probe was analyzed in triplicate, and specific signals were scored in relation to the signals of 2 housekeeping gene transcripts (GAPDH and cytochrome c). The results from different experiments were normalized to the expression of a calibrator probe, which was applied as a positive control in each experiment. An intron region of the interleukin-2 (IL-2) gene was always amplified to ascertain that the probes were not contaminated with genomic DNA. The primers used were assigned using the Universal ProbeLibrary Assay Design Center (http://www.roche-applied-science.com/shop/CategoryDisplay?catalogId=10001&tab=&identifier=Universal+Probe+Library&langId=-1).

### Histology and immunohistochemistry

The paws were fixed overnight in 4% paraformaldehyde, decalcified with 0.5*M* EDTA solution, and embedded in paraffin. Sections measuring 4 μm in thickness were analyzed. The extent of inflammation and cartilage damage in the paws was determined by morphometry as described elsewhere (13). Usually, 4 different sections per mouse sample were quantified from 3–4 mice for each time point. The inflamed area was defined as the infiltrated tissue area and was assessed in relation to the total tissue area (in toes, the area from the apex of a toe to the proximal tendon basis of the DIP joints was defined as the total area). Proteoglycan loss was determined as the percentage of destained cartilage area. For type II collagen detection, specimens were sequentially digested with hyaluronidase and proteinase K before incubation with the mouse anti–type II collagen antibody (Merck). For its quantification, the stained area was circumscribed and the relative stain intensity was determined using Photoshop software (Adobe Systems). All analyses were performed in a blinded manner by one of us (ER, TS, or VW). For immunohistochemistry, paraffin sections were dewaxed, blocked with 10% fetal bovine serum, and incubated with rabbit anti-S100A9 antibodies for 1 hour at room temperature. A Vectastain ABC-AP Kit (Vector) was used for visualization of the stained proteins.

### Statistical analysis

Mean ± SEM values were calculated, and statistical analysis was performed using the Mann-Whitney U test and GraphPad Prism software. *P* values less than 0.05 were considered significant.

## RESULTS

### Generation of doxycycline-inducible human TNFα–transgenic mice

The cDNA sequence for human TNFα was placed under the Tet-responsive promoter and introduced into the pronuclei of FVB/N fertilized mouse oocytes. The 3′-UTR was replaced by an SV40 poly(A) site to avoid a posttranscriptional regulation (see http://zmbe.uni-muenster.de/vwixler/retser.zip). Two founders, called *Tg_ihTNFαF8* and *Tg_ihTNFαF15*, were then crossed with the Tg_rtTA2S-M2 mice (15), kindly provided by Dr. John Strouboulis (Institute of Molecular Oncology, Biomedical Sciences Research Center, Varkiza, Greece). Southern blot analysis of genomic DNA from F1 hybrids revealed unique but different integration sites for the *Tg_ihTNFαF8* and *Tg_ihTNFαF15* founders, with 4 and 5 tandem transgenic copies, respectively (see http://zmbe.uni-muenster.de/vwixler/retser.zip). The F1 hybrid mice were further intercrossed to obtain mice homozygous for both transgenes. The offspring of the *Tg_ihTNFαF15* founder showed a typical Mendelian ratio and a normal lifespan, while those of the *Tg_ihTNFαF8* founder survived only as heterozygotes, further confirming the nonidentical incorporation sites of the human TNFα transgene in the genomes of these 2 founders. The heterozygous offspring of both founders showed no obvious abnormalities without addition of doxycycline, but upon treatment with the antibiotic, they developed a similar phenotype. Thus, we focused the present study on homozygous (*Tg_rtTA2S*^*%/%*^*/Tg_ihTNFαF15*^*%/%*^) mice, named doxycycline-inducible human TNFα–transgenic mice (also called ihTNFtg mice).

### Tissue expression pattern of human TNFα transcripts

**Figure 1:**
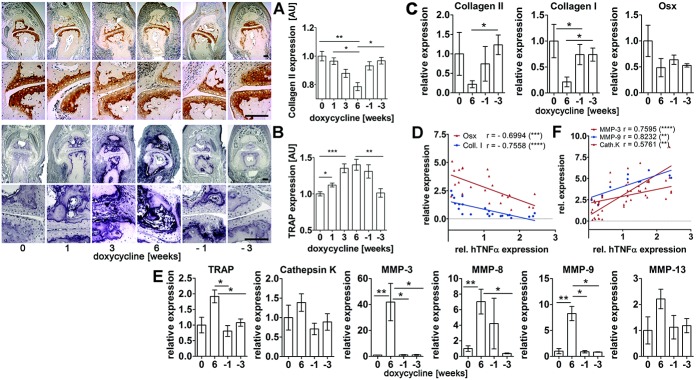
Expression of human tumor necrosis factor α (hTNFα) in doxycycline (Dox)–inducible human TNFα–transgenic mice after doxycycline administration. A and B, Systemic doxycycline-induced human TNFα (n = 8, 4, 9, and 6 mice for increasing concentrations shown) (A) and S100A8/A9 proteins (n = 6, 4, 5, and 6 mice for increasing concentrations shown) (B) in mouse serum, as determined by enzyme-linked immunosorbent assay. C, Transcriptional expression of human TNFα in tissues of untreated control mice and mice treated with doxycycline (0.5 mg/ml for 6 weeks), as analyzed by TaqMan quantitative reverse transcription–polymerase chain reaction. The amount of mRNA in fore paws of control mice was assigned a value of 1. Numbers of animals analyzed are shown above columns. D and E, Changes in body weight (D) and swelling of fore paws (E) during stimulation with various concentrations of doxycycline (n = 4 mice per time point for each group of mice). Values in A–E are the mean ± SEM. ∗∗ = *P* < 0.01; ∗∗∗ = *P* < 0.001 for doxycycline-treated mice versus untreated mice. F, Magnetic resonance imaging of fore paws before and after injection of contrast agent (CA; 0.5 mmoles/kg) (n = 5 mice per group). Arrowhead indicates prominent swelling of digits after doxycycline treatment. Bar = 1 mm. G, ^18^F-fluorodeoxyglucose (^18^F-FDG) positron emission tomography imaging showing locally increased ^18^F-FDG uptake as a surrogate marker for inflammatory activity in distal interphalangeal joints of doxycycline-treated (arrowheads) (n = 5 mice per experiment) but not untreated (control; n = 3 mice per experiment) doxycycline-inducible human TNFα–transgenic mice. In addition, a typical high uptake of the tracer by the heart and its excretion-based accumulation in the urinary bladder was always observed in both control and doxycycline-treated mice. Color figure can be viewed in the online issue, which is available at http://onlinelibrary.wiley.com/doi/10.1002/art.38026/abstract.

Doxycycline-inducible human TNFα–transgenic mice homozygous for both human TNFα and rtTA2S-M2 transgenes received different amounts of the antibiotic for 6 weeks, and the level of human TNFα in their sera was measured by ELISA in comparison with that in non–doxycycline-treated mice. Detectable levels of human TNFα protein were identified only in doxycycline-treated mice in a dose-dependent manner (Figure 1A), although a particular variation between single animals was observed as well. No soluble human TNFα cytokine could be detected in mice bearing only 1 of the transgenes (data not shown), confirming that the presence of both transgenes and stimulation with doxycycline are necessary for the expression of human TNFα.

The alarmins S100A8 and S100A9 are calcium-binding proteins that belong to the family of damage-associated molecular pattern molecules, and they are released by immigrating neutrophils and macrophages very early during inflammation. They are increasingly used as biomarkers for ongoing inflammatory processes, especially for RA (16,17). The amount of S100A8/A9 heterodimer used as a biomarker to monitor inflammation was increased in the sera of the mice treated with doxycycline, and the degree of elevation was dependent on the amount of doxycycline the mice received (Figure 1B), suggesting that ongoing inflammation takes place in doxycycline-treated mice and that the induced human TNFα is functionally active.

To study the expression pattern of the human TNFα transgene, different organs from doxycycline-stimulated mice were analyzed by TaqMan qRT-PCR in comparison to different organs from untreated mice. Upon administration of doxycycline, the synthesis of human TNFα mRNA was switched on in several organs, with fore and hind paws showing the highest induction, followed by skin, thymus, and lung (Figure 1C). The degree of induction varied between individuals; however, the detected amounts of human TNFα mRNA, including the low scores, were specific and not due to contamination of the RNA samples with genomic DNA. The qRT-PCR counts for an intron sequence of the IL-2 gene were usually at least 2 orders of magnitude lower than those of the TNFα signals (data not shown). The nontransgenic C57BL/6 wild-type mice treated with doxycycline did not express any human TNFα mRNA, and the established double-transgenic mouse line (doxycycline-inducible human TNFα–transgenic mice) showed only negligible levels of human TNFα mRNA when kept untreated.

### Doxycycline-inducible human TNFα–transgenic mice develop polyarthritis

**Figure 2:**
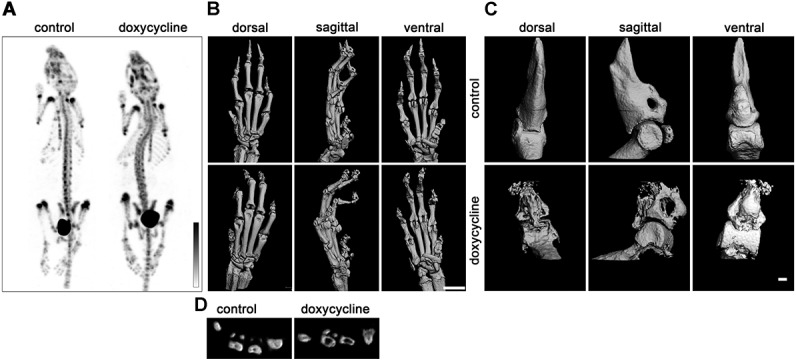
Inflammation in paws of doxycycline-inducible human tumor necrosis factor α (hTNFα)–transgenic mice is reversible. Six-week-old doxycycline-inducible human TNFα–transgenic mice received water with 0.5 mg/ml doxycycline for 6 weeks, after which they received water without the antibiotic (weeks labeled −1 to −5). A, Changes in grip strength of hind paws during doxycycline treatment (n = 4 mice for each time point). B, Alteration in transcription of the human TNFα transgene in paws (n = 5, 5, 4, and 4 mice for the respective time points shown). C, Levels of soluble human TNFα cytokine in serum (n = 4–9 mice at each time point). D, Fore paws of doxycycline-inducible human TNFα–transgenic mice before (0 weeks), during (6 weeks), and after (–5 weeks) doxycycline treatment. E, Histologic changes in toes of hind paws. Images of proximal interphalangeal (PIP) joints are shown (toluidine blue staining). Note the progression of synovitis (asterisks), cartilage destaining (arrowheads), and attachment and invasion of pannus-like tissue into bone (hatchmark). Bars = 200 μm. F and G, Histomorphometric quantification of inflamed synovium (F) and cartilage loss of proteoglycan (G) in PIP joints shown in E (n = 4 mice for each time point). Values are the mean ± SEM. ∗ = *P* < 0.05; ∗∗ = *P* < 0.01; ∗∗∗ = *P* < 0.001.

Long-term expression of high TNFα levels is associated with the development of chronic inflammatory diseases, with RA being one of the most prominent TNFα-mediated disorders (10,12,18). As the progression of arthritis is usually accompanied by body wasting, swelling of joints, and movement impairment, we analyzed the alteration of these parameters in doxycycline-inducible human TNFα–transgenic mice during doxycycline application (Figures 1D and E and 2A). Mice bearing only 1 transgene (either the human TNFα transgene or the rtTA2S-M2 transgene) as well as untreated double-transgenic mice did not show any alterations. However, doxycycline-inducible human TNFα–transgenic mice showed the first macroscopic pathologies as early as 2 weeks after doxycycline administration. Swelling and redness of the fore and hind paws along with diminished grip strength and reduced mobility appeared at that time and progressed constantly with continuous doxycycline application. The severity of the symptoms correlated with the amount of antibiotic applied, as did human TNFα levels in sera.

Interestingly, ankle joints were not affected, unlike the case with already-described human TNF–transgenic mice with constitutive expression of the cytokine (10,12), but the digits were strongly affected, and fore paws were more strongly affected than hind paws. Strong joint swelling and formation of so-called “sausage digits” with almost characteristic involvement of the DIP joints and nail deformation were very prominent. MR imaging of fore paws at a spatial resolution of 52 μm revealed extensive swelling of the digits and edema of all soft tissues in doxycycline-treated mice. After contrast agent application (Figure 1F), all soft tissues exhibited higher signal intensity. The observed features resembled to a certain degree the signs of PsA. However, we observed no plaque formation on the skin (which often accompanies psoriasis), either on the digits or on other parts of the body, although skin irritation with hair loss on the head, neck, or ventral side often appeared (see http://zmbe.uni-muenster.de/vwixler/retser.zip). No other gross alterations or organ inflammation could be detected when a whole-body PET imaging study was performed using ^18^F-FDG (Figure 1G).

### Development of arthritis in doxycycline-inducible human TNFα–transgenic mice is reversible

To ascertain whether the observed phenotype was reversible, mice were stimulated for 6 weeks with 0.5 mg/ml doxycycline, after which the antibiotic was removed. The grip strength improved continuously after the antibiotic was withdrawn (Figure 2A). Remarkably, 1 week after antibiotic withdrawal, transcription of human TNFα was barely detectable in the paws and human TNFα protein was barely detectable in the sera of doxycycline-inducible human TNFα–transgenic mice (Figures 2B and C). While serum levels of human TNFα varied from 100 pg/ml to 1,300 pg/ml in mice treated for 6 weeks, only 1 of the analyzed animals expressed fully 42 pg/ml human TNFα 1 week after removal of the antibiotic. Consistent with these data, macroscopic paw symptoms also improved. Digit swelling was visibly reduced after doxycycline removal, and grip strength increased progressively; after 3–5 weeks even screwed or broken nails had recovered (Figure 2D).

We next performed histologic analyses of inflamed paws, concentrating our attention on the DIP joints. Toluidine blue staining of paw sections revealed the first signs of abnormalities as early as 1 week after doxycycline treatment. Hyperplasia of synovial tissue and infiltration by polymorphonuclear and lymphoid cells into soft tissue and the joint space as well as cartilage destruction (indicated by the destained proteoglycan area) were evident. Particularly distinguishing, however, was the high amount of pannus tissue, which grew progressively with time and penetrated largely into the bone tissue (Figure 2E). Morphometric quantification of such inflammation and cartilage destaining showed that these features increased with the duration of doxycycline stimulation (Figures 2F and G). However, withdrawing the antibiotic stopped the progression of these abnormalities and induced gradual improvement, which resulted in almost complete recovery 3 weeks later.

### DIP joints but not ankle joints are predominantly affected

**Figure 3:**
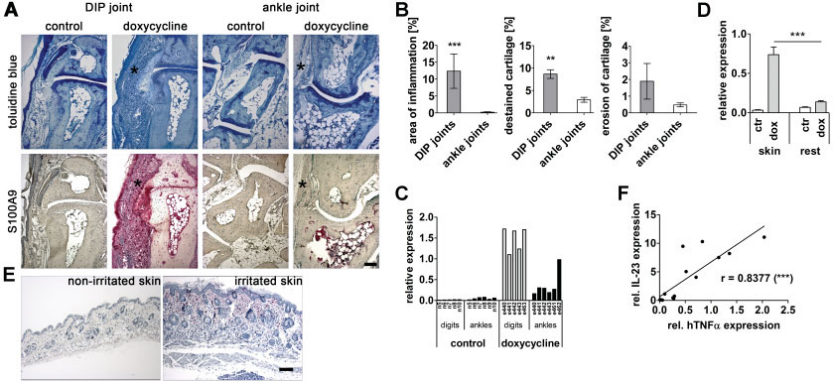
Distal interphalangeal (DIP) joints but not ankle joints are severely inflamed in doxycycline-inducible human TNFα–transgenic mice after stimulation with doxycycline. Doxycycline-inducible human TNFα–transgenic mice were treated with 0.5 mg/ml doxycycline for 6 weeks or left untreated (control). A, Staining of serial sections of hind paws with toluidine blue (top) or antibodies to S100A9 protein (red) (bottom). Asterisks indicate a strong synovitis in DIP joints but not ankle joints of doxycycline-treated mice. B, Quantification of inflammation (left), cartilage destaining (middle), and cartilage erosion (right) by morphometry (n = 4 mice per group). ∗∗ = *P* < 0.01; ∗∗∗ = *P* < 0.001 versus ankle joints. C, Expression of human TNFα transcripts in hind paw digits and ankles, quantified by TaqMan quantitative reverse transcription–polymerase chain reaction (RT-PCR). Values for separate numbered animals are presented. D, Quantitative RT-PCR analysis of human TNFα expression in the skin and the rest of the tissues of hind paws (n = 5 mice per group). ∗∗∗ = *P* < 0.001. E, S100A9 staining (red) of a healthy skin section and an irritated (see http://zmbe.uni-muenster.de/vwixler/retser.zip) skin section from a doxycycline-treated mouse. Nuclei (blue) were counterstained with hematoxylin. F, Relationship between human TNFα and interleukin-23 (IL-23) mRNA transcripts in skin samples from doxycycline-treated mice (n = 12 mice). ∗∗∗ = *P* < 0.001. Bars = 100 μm. Values in B and D are the mean ± SEM. See Figure 1 for other definitions.

In transgenic mice constitutively expressing TNFα, ankle joints, but not digit joints, were primarily affected (4,11). Therefore, we performed a direct comparison of both joint types of the same hind foot. Consistent with macroscopic observations, the toes were more severely affected than the ankles, and the DIP joints showed the greatest pathologic changes (Figure 3A). We measured 2–3-fold less cartilage destruction and erosion and 5–8-fold less pannus area in ankle joints than in DIP joints (Figure 3B). Staining for S100A9 protein as a marker of inflammation further confirmed that the major inflammation process occurred in the distal toes but not in the ankle regions of hind paws, as only weak staining for S100A9 was seen in the ankle joint area (Figure 3A).

**Figure 4:**
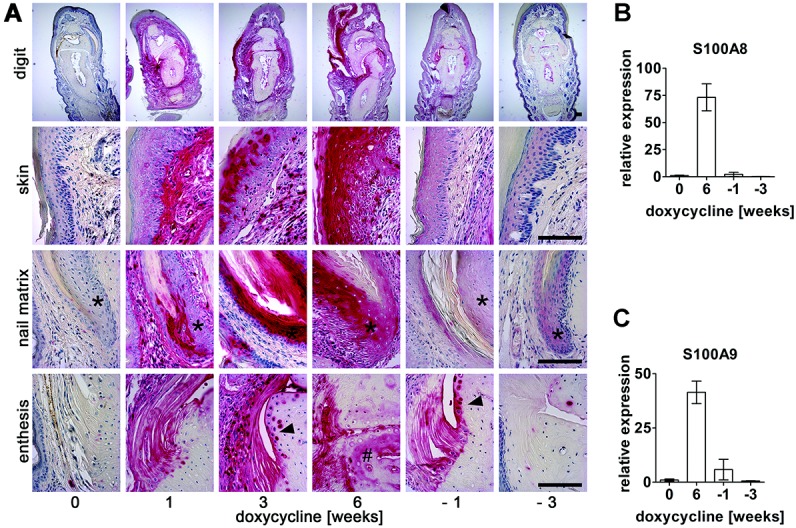
Inflammation in the toes of doxycycline-inducible human tumor necrosis factor α–transgenic mice during (0–6 weeks) and after (weeks labeled −1 to −3) doxycycline treatment, analyzed by S100A9 (red) expression. A, Immunohistochemistry of S100A9. Representative images of S100A9-stained sections of hind paw toes are depicted. Note the inflammation of nail matrix (asterisks), chondrocytes (arrowheads), and bone tissue (hatchmark). Bars = 100 μm. Samples were counterstained with hematoxylin to reveal nuclei (blue). Three to 5 animals per each time point were analyzed. B and C, Changes in S100A8 (B) and S100A9 (C) transcripts in paws during doxycycline treatment, determined by TaqMan quantitative reverse transcription–polymerase chain reaction (n = 5 mice for each time point). Mean mRNA levels in untreated mice were assigned a value of 1. Values are the mean ± SEM.

It is conceivable that the observed differences between digits and ankles are based on different expression levels of human TNFα in these organs. To assess this possibility, we compared the expression of the human cytokine in toes and ankles by qRT-PCR. As shown in Figure 3C, digits indeed expressed much higher levels of human TNFα than did ankle structures, and the induced cytokine was found predominantly in the skin samples of paws but not in the rest of the tissues (Figure 3D). The high expression of human TNFα in the skin (Figures 1C and 3D) suggests an ongoing inflammatory disorder in the organ, despite the absence of gross macroscopic abnormalities. Indeed, the histologic analysis not only of the digit skin (Figures 3A and 4A), but also of the irritated dorsal skin distal from toes (Figure 3E) showed clear signs of inflammation on doxycycline induction, and the amount of human TNFα mRNA induced in dorsal, abdominal, or paw skin samples correlated highly with levels of the cytokine IL-23 (Figure 3F), which is typically up-regulated during psoriasis (19). The doxycycline per se, however, had only a minor, if any, effect on the development of dermal inflammation in nontransgenic mice (see http://zmbe.uni-muenster.de/vwixler/retser.zip). It is worth mentioning that besides the skin and paws, all other tissues and joints analyzed, including the gut and the highly “arthritis-sensitive” joints, such as the spine, sacroiliac, and hip joints, were S100A9 negative and did not show noteworthy morphologic alterations (see http://zmbe.uni-muenster.de/vwixler/retser.zip).

### Doxycycline-inducible human TNFα–transgenic mice develop onychia, psoriasis, and enthesitis

To further define the structures in which inflammation takes place in the DIP joints, paw sections of mice stimulated for different amounts of time with doxycycline were stained for S100A9 protein. While no staining was observed in doxycycline-untreated mice, strong S100A9 staining was present as early as 1 week after doxycycline addition. The staining intensity rose further and remained during the whole time of doxycycline stimulation. However, it dropped very quickly when the antibiotic was removed (Figure 4A). The alteration in S100A9 staining in paws correlated very well with transcriptional induction of both S100A8 and S100A9 genes (Figures 4B and C). Taken together, these data are consistent with the morphometric analysis of inflammation and cartilage destruction shown in Figure 2. Further, consistent with the results presented in Figures 2 and 3, only the phalanges showed strong staining for S100A9 protein, with the DIP joint areas being the most severely affected. Indeed, the more distant the joints from the tips of the digits, the less S100A9 staining was noted.

Among stained structures, skin, nail matrix, and entheses were the most protruding (Figure 4A). Given that a positive S100A9 signal indicates where inflammation takes place, induction of dermal inflammation, onychia, and enthesitis of digit joints and especially of DIP joints was most obvious. Again, the inflammation of these organs was reversible, as it declined to the control level shortly after ending doxycycline treatment, indicating that despite the strong induction of human TNFα, the disease did not turn into a chronic state with high expression of endogenous inflammatory cytokines. Interestingly, keratinocytes of both the skin and nail matrix were the cells with the highest expression of S100A9 protein. A serial-sections staining for S100A9 and pan-keratin confirmed this observation (see http://zmbe.uni-muenster.de/vwixler/retser.zip) and further showed that not the actively proliferating parts of keratinocyte sheets, but the more differentiated spinous layers were highly positive for S100A9 protein. The staining was seen very early after TNFα induction and increased further with ongoing doxycycline stimulation, indicating an augmentation of inflammation in these tissues. Further, the activation of nail matrix keratinocytes seemed to occur earlier and more intensely than the activation of skin keratinocytes. While only single cells of the epidermis were weakly S100A9 positive 1 week after doxycycline administration, many cells of the nail root always showed strong S100A9 staining at this time. The pattern of arthritis and skin involvement somehow reflects a psoriatic phenotype, although typical signs, such as epidermal hyperproliferation, are missing.

Next, we aimed to analyze how quickly the signs of inflammation could be recorded after doxycycline administration. Therefore, we analyzed DIP joints as early as 24 hours after doxycycline treatment (see http://zmbe.uni-muenster.de/vwixler/retser.zip). The soft tissue around DIP joints was already S100A9 positive at that time, with the released soluble protein being captured by acellular structures of the connective tissue. Ligaments and bone tissue were stained weakly, as were also a few keratinocytes. It was striking, however, that essentially no cell infiltrates, including S100A9-positive cells, were present on day 1 of doxycycline administration. This did not change markedly on day 3 of doxycycline administration, but from day 7 onward, numerous infiltrating immune cells, including S100A9-positive cells, were noted (Figures 2E and 4A) (see http://zmbe.uni-muenster.de/vwixler/retser.zip). Taken together, these data show that the S100A9 protein is exposed in distal phalangeal tissue very early after the induction of TNFα; it precedes the immigration of immune cells and already reflects the early beginnings of inflammatory activities in these tissues.

### Cartilage and bone phenotype

**Figure 5:**
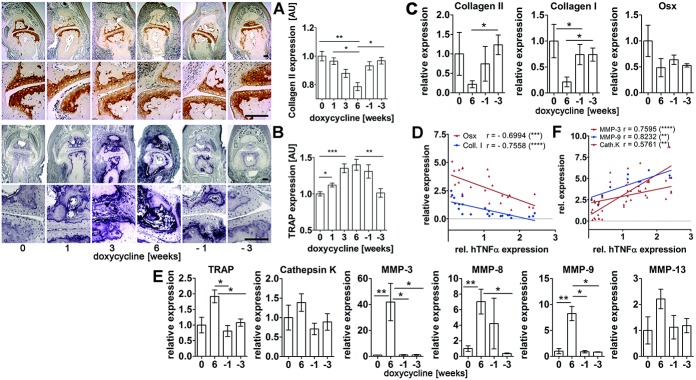
Alterations in gene expression are reversible. A and B, Immunostaining (left) and morphometric quantification (right) of type II collagen (top) and tartrate-resistant acid phosphatase (TRAP) (bottom) in distal interphalangeal joints during (0–6 weeks) and after (weeks labeled −1 to −3) doxycycline stimulation (n = 4 mice per group). Bars = 100 μm. C and E, Transcriptional alterations of the indicated genes in paws of doxycycline-inducible human tumor necrosis factor α (hTNFα)–transgenic mice (n = 9 paws per group). Values are the mean ± SEM. D and F, Relationship between human TNFα and indicated mRNAs in paws of doxycycline-inducible human TNFα–transgenic mice treated for 6 weeks with doxycycline (n = 23 paws). Mean mRNA levels in untreated mice were assigned a value of 1. Osx = osterix; Coll. I = type I collagen; MMP-3 = matrix metalloproteinase 3; Cath. K = cathepsin K. ∗ = *P* < 0.05; ∗∗ = *P* < 0.01; ∗∗∗ = *P* < 0.001; ∗∗∗∗ = *P* < 0.0001.

An additional peculiarity was the S100A9 staining of the chondrocytes and bones of the phalanges from week 1 onward as well as the bone-invading pannus-like tissue at later stages (Figures 3A and 4A) (see http://zmbe.uni-muenster.de/vwixler/retser.zip). As for other S100A9 stainings, these also disappeared almost completely 3 weeks after the withdrawal of doxycycline (Figure 4A). To study whether the observed cartilage damage in DIP joints was restricted to the loss of proteoglycans alone or was accompanied by additional tissue loss, we analyzed changes in expression of the articular cartilage matrix protein type II collagen by immunohistochemistry and qRT-PCR (Figures 5A and C). Quantification of type II collagen staining revealed a gradual reduction in the DIP joints during doxycycline stimulation and a quick and significant recovery after doxycycline removal. Along with the protein loss, the transcriptional activity of the type II collagen gene decreased nearly 5-fold at week 6 but recovered when doxycycline was withdrawn. To see whether osteoblast function was impaired with doxycycline treatment as well, we analyzed the transcription of 2 well-established markers of osteoblast activity, type I collagen and osterix. The mRNA levels of both genes, and especially of type I collagen, were strongly reduced with doxycycline stimulation, and similar to type II collagen, they were restored when the antibiotic was withdrawn (Figure 5C). Further, consistent with numerous data showing that TNFα suppresses the expression of type I collagen and osterix (20,21), the transcript levels of both genes showed a high inverse correlation with the amount of induced human TNFα in the organ (Figure 5D).

Development of both PsA and RA is frequently accompanied by bone erosion due to osteoclast activation and increased synthesis of collagenolytic proteinases (22,23). Morphometric analysis of tartrate-resistant acid phosphatase (TRAP)–positive tissue in the distal phalanges as a marker of activated osteoclasts and monocytes showed a clear increase in TRAP expression during the time of doxycycline treatment, and as with other signs of TNFα-mediated inflammation in this organ, TRAP expression reverted to control levels after removal of the antibiotic (Figure 5B). Also, the transcriptional activity of TRAP, cathepsin K, and several MMPs increased with doxycycline stimulation and declined with its withdrawal (Figure 5E). An especially high transcriptional increase was noted for MMPs 3 and 9, and mRNA levels of these genes as well as those of cathepsin K in single animals correlated very well with mRNA levels of human TNFα (Figure 5F). Thus, in addition to severe inflammation, our analyses of the toes also revealed cartilage destruction and signs of bone erosion due to altered activation of osteoblasts and osteoclasts.

**Figure 6:**
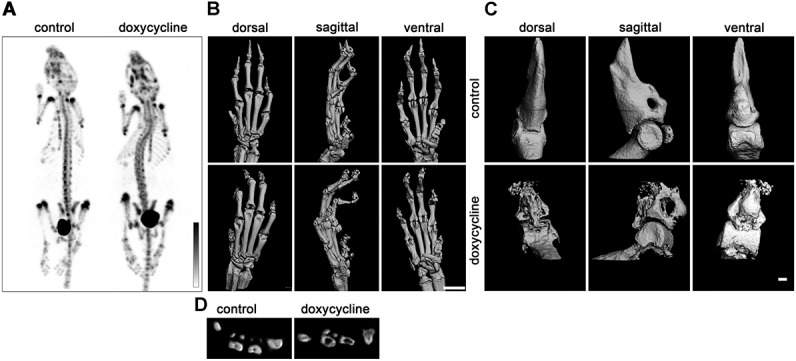
Bone phenotype of control (unstimulated) doxycycline-inducible human tumor necrosis factor α (TNFα)–transgenic mice and doxycycline-inducible human TNFα–transgenic mice treated with doxycycline for 6 weeks. A, In vivo methylene diphosphonate (MDP) single-photon–emission computed tomography images showing a similar distribution pattern of MDP in doxycycline-treated (n = 3) and control (n = 2) doxycycline-inducible human TNFα–transgenic mice. B, Representative micro–computed tomography (micro-CT) images of fore paws of control (n = 2) and doxycycline-treated (n = 4) doxycycline-inducible human TNFα–transgenic mice. Bar = 1 mm. C, Representative micro-CT images of the first 2 distal phalanges of the middle digits. Bar = 100 μm. D, Cross-section of metacarpals.

Visual inspection of in vivo SPECT images did not reveal gross alterations of skeleton structures (Figure 6A). Therefore, we performed a comparative micro-CT analysis of the fore paws from control and doxycycline-treated mice to explore whether bone tissue damage takes place in inflamed toes (Figures 6B and C). Bones of doxycycline-treated mice, but not those of control doxycycline-inducible human TNFα–transgenic mice, displayed clear signs of tissue damage. Especially bones of the first 2 distal phalanges were severely affected, exactly where the major inflammation was always seen. However, clear signs of bone resorption were also detectable in more distal bones (Figure 6D). These results are consistent with data shown in Figure 5 and suggest an altered osteoblast/osteoclast homeostasis in paws with human TNFα induction.

## DISCUSSION

While several TNFα-transgenic mice already exist, no inducible mouse model has previously been described. In the present study, we describe the phenotype of such a mouse line. We show that human TNFα expression is present in the blood, skin, and fore paws of doxycycline-inducible human TNFα–transgenic mice exclusively with doxycycline administration and is reduced to control levels as early as 1 week after doxycycline removal. We further show that upon doxycycline stimulation, doxycycline-inducible human TNFα–transgenic mice develop a wide range of abnormalities that are characteristic of arthritis-like diseases. Inflammation of distal phalanges accompanied cartilage and bone destruction and dermal inflammation. However, when doxycycline was removed, almost all abnormalities were reversed during the following 3 weeks. While human TNFα was expressed in many organs and reached high levels in the blood that were comparable with those reported in Tg197-transgenic mice (10), only the digits and, to a lesser extent, the skin and ankles were affected in doxycycline-inducible human TNFα–transgenic mice. We have not seen signs of inflammation in other organs, either by macroscopic or histologic analyses. Much higher local than systemic concentrations of human TNFα might be a reasonable explanation for this.

A major advantage of our mouse model is that the disease is inducible and can be induced at any stage of life, and the degree as well as the state of inflammation (acute, chronic, or batchwise) can be fine-tuned. Further, doxycycline-inducible human TNFα–transgenic mice not yet exposed to doxycycline are vital and fertile and can therefore be crossed, without limitations, with other transgenic or knockout mice, and we believe that the doxycycline-inducible human TNFα–transgenic mouse is a valuable tool for studying the intrinsic mechanisms of TNFα-mediated diseases.

## AUTHOR CONTRIBUTIONS

All authors were involved in drafting the article or revising it critically for important intellectual content, and all authors approved the final version to be published. Dr. Wixler had full access to all of the data in the study and takes responsibility for the integrity of the data and the accuracy of the data analysis.

**Study conception and design.** Retser, Schied, Skryabin, Vogl, Kanczler, Hamann, Niehoff, Hermann, Eisenblätter, Wachsmuth, Pap, van Lent, Loser, Roth, Zaucke, Ludwig, Wixler.

**Acquisition of data.** Retser, Schied, Skryabin, Vogl, Kanczler, Hamann, Niehoff, Hermann, Eisenblätter, Wachsmuth, van Lent, Roth, Zaucke, Wixler.

**Analysis and interpretation of data.** Retser, Schied, Skryabin, Vogl, Kanczler, Hamann, Niehoff, Hermann, Eisenblätter, Wachsmuth, Pap, van Lent, Loser, Roth, Zaucke, Ludwig, Wixler.
